# Western Hemisphere Zuphiini: descriptions of *Coarazuphium whiteheadi*, new species, and *Zuphioides*, new genus, and classification of the genera (Coleoptera, Carabidae)

**DOI:** 10.3897/zookeys.315.5293

**Published:** 2013-07-04

**Authors:** George E. Ball, Danny Shpeley

**Affiliations:** 1 Department of Biological Sciences, University of Alberta, Edmonton, Alberta T5G 2E9, Canada

**Keywords:** Taxonomy, Zuphiitae, classification, key, anophthalmy, phylogenetic relationships, brachyptery, cave inhabitants, laboulbenealian fungi

## Abstract

Based on small samples (exemplars) analyzed with morphological methods, including detailed descriptions and illustrations, this study treats primarily the *Zuphium* genus-group in the Western Hemisphere, which comprises two precinctive genera: *Coarazuphium* Gnaspini, Vanin & Godoy, 1998 (type species *Parazuphium tessai* Godoy & Vanin, 1990) and *Zuphioides* **gen. n.** (type species *Zuphium mexicanum* Chaudoir, 1863). The genus *Coarazuphium* includes six troglobitic species from Brazilian caves, and one probably hypogaeic (troglophilic) species from the mountains of Oaxaca, in Mexico (*Coarazuphium whiteheadi*, **sp. n.**, type locality, ridge top, in western Oaxaca, Mexico, at 2164 m, 35 km north of San Pedro Juchatengo, 16.462N, 97.010W). The epigaeic genus *Zuphioides* includes 23 species, with its geographical range extended from Neotropical temperate Argentina in southern South America, northward through the tropics to north temperate southeastern Canada, in the Nearctic Region. Keys are provided to the species of *Coarazuphium* and to thegenera of Western Hemisphere Zuphiini.

## Introduction

**Map 1.**
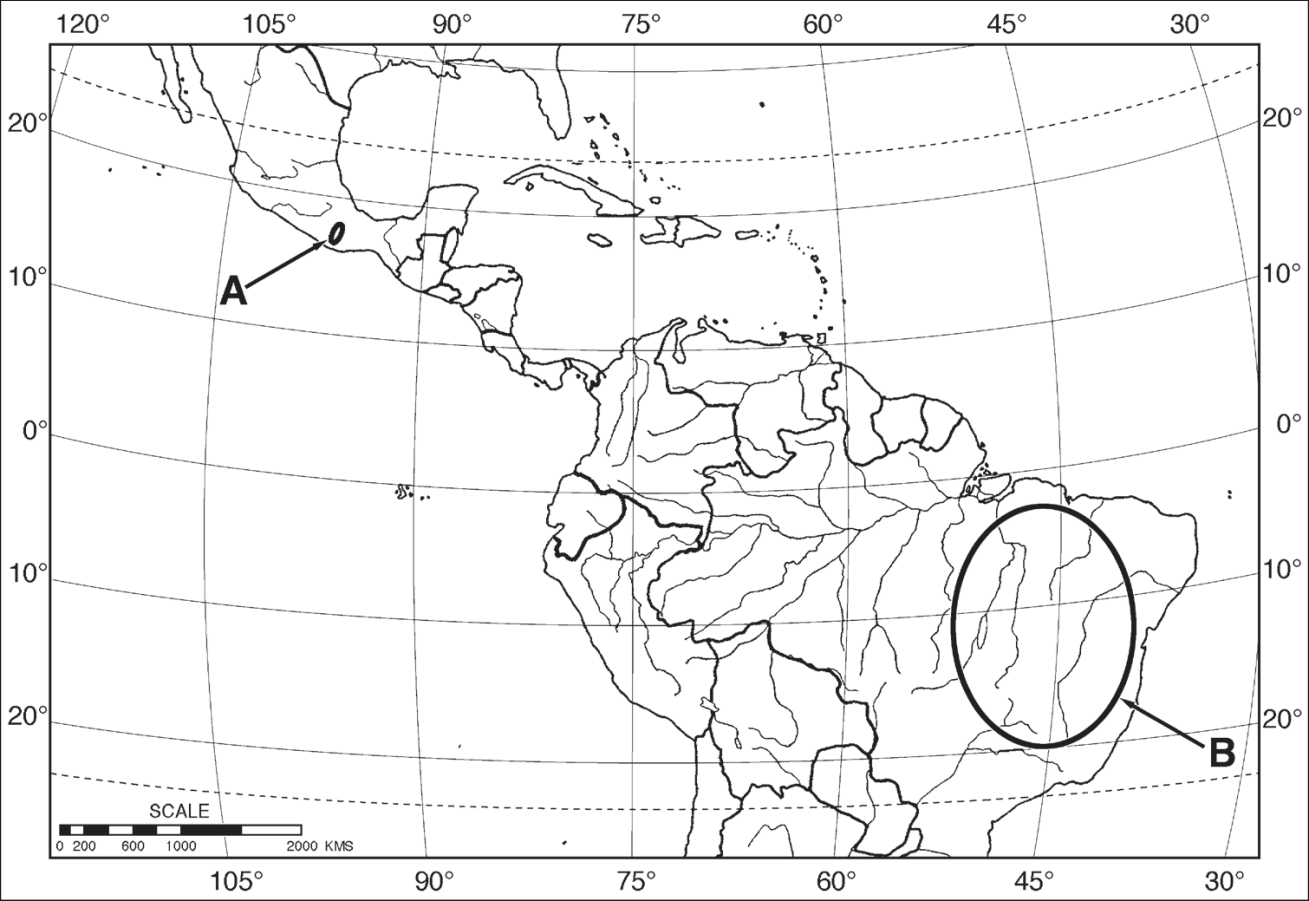
Outline map of southern North America, Middle America and northern South America, showing generalized geographical range of species of *Coarazuphium*. **A** *Coarazuphium whiteheadi*, new species **B** *Coarazuphium bezerra*, *cessaima*, *formoso*, *pains*, *tapiaguassu* and *tessai*.

The taxonomic investigation reported herein, began with discovery of an undescribed Mexican species of *Coarazuphium* Gnaspini, Vanin & Godoy. Considering that *Coarazuphium* was known previously only from caves in eastern Brazil, its known range extension into the northern part of Middle America (Map 1) was reason to examine it closely, with the expectation of broadening our concept of the genus, and perhaps even of its tribe, the Zuphiini.

Although the six known Brazilian species had been clearly described and well illustrated, a more detailed comparison of *Coarazuphium* with other zuphiines seemed desirable. Closer comparisons with the geographically widespread, morphologically similar *Zuphium* of authors were undertaken, beginning with a sampling of the Western Hemisphere species, and extending into the Eastern Hemisphere taxa, principally those from Western Europe and eastern Africa, with emphasis on the wide-ranging *olens* species group.

We broadened our study by placing *Coarazuphium* and *Zuphioides* (see below) in the context of a short review, based on the literature, of the Western Hemisphere zuphiine genera. We also took into account the classification of the Zuphiini (see Appendix I).

## Material, methods and terms

### Material

This study is based on examination of 94 specimens of Zuphiina, from the Western Hemisphere Nearctic and Neotropical Regions, and Eastern Hemisphere Palaearctic, Afrotropical, and Oriental Regions. Much of the material was in the Strickland Museum, Department of Biological Sciences, University of Alberta (UASM). Through the generosity of Terry L. Erwin, a sampling of zuphiines was received from the Department of Entomology, United States National Museum of Natural History, Smithsonian Institution, Washington, D. C., U.S.A. 20560 (USNM).

### Methods

Measurements. Measurements were made with an ocular micrometer in a Wild M5 stereoscopic microscope, at 50×. Measurements of external body parts and abbreviations used for them in the text are:

HL Length of head - linear distance from apex of extended left mandible to posterior margin of the postocciput;

HW Width of head - maximum distance across head, including eyes;

A1L Length of antennomere 1 - linear distance from base of antennomere 1 to apex of antennomere 1;

A2-4L Length of antennomeres 2–4 - linear distance from base of antennomere 2 to apex of antennomere 4;

PL Length of pronotum - linear distance from anterior to posterior margin, measured along the midline;

PWM Maximum width of pronotum - greatest linear transverse distance;

EL Length of elytra - linear distance from humerus to apex;

EW Width of elytra - maximum distance across the elytra.

OBL Overall Body Length is the sum of HL, PL, and EL. Values for ratios for species were computed, using the measurements above: A1L/A2-4L; HW/PW. These numerical data are illustrative rather than definitive.

To express quantitatively proportions of the phallus, three measurements were made, using left lateral and dorsal aspects as illustrated in Figs 10 and 13:

PL Length of phallus - measured in a straight line from basal to apical margin;

PAL Length of apical portion - measured in a straight line from apical margin of periostial area to apical margin;

PSW Width of phallus - maximum transverse distance across the shaft, in ventral aspect.

These measurements were combined as two ratios PAL/PL; and PSW/PL. These numerical data are illustrative rather than definitive.

Preparation of material. Dissections were made by using standard techniques. Genitalia and other small structures were preserved in glycerine in microvials and pinned beneath the specimens from which they were removed. Larger structures and those that were gold-coated for study with the SEM were glued to cards pinned beneath the specimens from which they were removed.

Micrographs of isolated structures were taken with a JEOL JSM 6301 FXV field emission SEM. Line drawings of selected body parts were prepared by using a camera lucida on a Wild W5 stereoscopic microscope. Plates were prepared by using Adobe Photoshop CS 4.

Citation of figures. Figures included in the present publication are cited in the text as “Fig.” Those previously published are cited “fig.”

Label data. For type material, the information on each label is reproduced as exactly as is possible using ordinary type. Information on each label is enclosed in quotation marks; as well, a semicolon marks the end of a label. A slash mark (/) indicates the end of each line of text.

### Terms Structural features

Most of the terms used to designate details of structures are found in textbooks of general entomology, or are used by coleopterists, generally. Other words, used to designate particular structures or parts thereof, are not in general use, though they have been used by us in previous publications. We provide information about these words here, as well as names that have been changed for certain structural features.

Microsculpture. A “sculpticell” is the space on the surface of the cuticle enclosed by adjacent microlines of the integumental system of microsculpture (Allen and Ball 1980: 485-486). In most groups, overall, microsculpture is varied. In contrast, in the *Zuphium* speciesgroup, it is absent (surface smooth) or simply isodiametric, with microlines very fine and sculpticells flat to slightly granulate. Emphasis is placed on description of microsculpture of the sclerites of the dorsal surface, which is adequate for characterization of the taxa of the *Zuphium* genus-group.

Chaetotaxy. This term refers to the so-called “fixed setae”, which are the long, evidently tactile, commonly encountered setae on carabids: dorsal labral; clypeal; supraorbital; stipital; submental; mental; glossal; palpigeral; pronotal; elytral parascutellar, discal, and umbilicate (or lateral); coxal, trochanteral, femoral, and tarsomeral; abdominal sternal ambulatory (sterna IV, V, VI); and abdominal sternal terminal (sternum VII, near posterior margin). Standard leg setae were not included because of difficulty in distinguishing them from the general body setation; but see Pellegrini and Ferreira (2011a, 2011b).

Body parts. The term “segment” is restricted for use to those body parts that reflect embryonic somites; thus, somite-like portions of the abdomen are referred to as segments. Abdominal segments are designated by Roman numerals corresponding to their respective somites. The first complete sternum is III, and the last one normally exposed is VII. For numbering the genital somites, we follow Bils (1976).

Head. The term “head capsule” in the *Zuphium* genus-group is restricted to that portion of the head that extends from the anterior margin of the clypeus to the broad part of the occiput that precedes the more or markedly narrowed posterior part of occiput + postocciput.

Eyes. Three conditions are recognized in the *Zuphium* genus-group: normal size (macrophthalmous), readily seen, convex, with distinct ommatidia (Fig. 3B); small (microphthalmous), virtually flat, without ommatidia, not readily seen (Fig. 3A); and absent (anophthalmous).

Antennae. Antennomere 1 (Figs 4B, 4D) exhibits two or three types of trichoid setae, based on size and declination: **as1**, single, erect, long; **as2**, row of several, erect, moderately long; and **as3**, more or less numerous, short, decumbent.

Mandibles. Shpeley and Ball (2001: 9–21, figs 3A–E) characterized the mandibles of the lebiine subtribe Pericalina, and illustrated the major features with SEM figures. We use the same system here. See Figs 6A–F and 7A–F.

Labium. The labium of the *Zuphium* genus-group is standard for Carabidae. For the combined glossae and paraglossae, we use the standard term ligula. The central sclerotized, apically setigerous, structure is the glossal sclerite. See Figs 8C and 8F.

Male tarsal vestiture. Two types of adhesive vestiture on the ventral surface of the fore tarsi are exhibited by males of the *Zuphium* genus-group: articulo-setae (Fig. 9A, **as**) and biseriate squamo-setae (See Stork 1980, for a thorough discussion of this topic).

Male genitalia. Based on economy of expression, in preference to the widely used terms “median lobe” and “internal sac”, we use “phallus” and “endophallus”, respectively. Such usage is also well established in the entomological literature treating the male reproductive system. The surface of the phallus treated by convention as dorsal is really the ventral surface, and vice versa (Deuve 1993: 88). As in our previous publications, we have chosen to remain with the conventional usage. Phalli were classified as anopic, with the ostium dorso-medial. The phallus (e.g., Figs 10A–10F, and 13A–13C), illustrated in left lateral, ventral and right lateral aspects, with base toward the bottom of the page, exhibits interspecific differences in form. These differences are seen readily as overall patterns (‘Gestalt’) but are not so easily described, except with notation of differences in size and shape of the apical area. To provide the basis for verbal description, two principal regions are distinguished-- the shaft (**s**), and basal bulb (**bb**), or lobe, or phallobase. The latter is the swollen area set at an angle to the ventrally curved shaft, surrounding the basal opening; the periostial membrane (**om**), which surrounds the ostium, marks the place of egress of the endophallus during copulation; an apical portion (**ap**) or phalloapex, extends distally from the apex of the periostial membrane to the apex of the phallus. The phalli in lateral aspect exhibit curvature ventrally. A pair of slender paraostial sclerites (**ps**) is evident (Figs 13A, 13C) or not (Fig. 10A).

Ovipositor (Figs 11A–11F) andfemale genital tract (Figs 12A, 12B and 13D). For naming the sclerites of the adephagan ovipositor and units of the female genital tract, we follow the system used by Liebherr and Will (1998; see figures therein). For the gonocoxites (**gc1**, **gc2**), the surfaces that are ventral in the infolded position are lateral when the ovipositor is extended; thus such surfaces are designated as lateral, and the other surfaces are designated accordingly.

## Systematics

Some of the morphological features that distinguish *Coarazuphium* from the Eastern Hemisphere widespread *Zuphium olens* species group (includes *Zuphium olens* (Rossi, 1790), type species of the genus) are shared with the Western Hemisphere species of *Zuphium* of authors (Table 1). From that distribution of character states, we conclude that the Western Hemisphere *Zuphium* of authorsforms an assemblage different from *Coarazuphium* andfromthe *Zuphium olens* species group, and this bridges the morphological gap between those two taxa. A reasonable taxonomic expression of this situation seems to us is to recognize three groups of generic rank: *Zuphium* (*sensu stricto*), *Zuphioides* **new genus** (= *Zuphium* of auothors), and *Coarazuphium*.

**Table 1.** Comparison of diagnostic character state combinations exhibited by exemplars of *Zuphium* (*sensu stricto*), *Zuphioides*, new genus, and *Coarazuphium* Gnaspini, Vanin & Godoy.<br/>

		**Taxa and character states**		
**No.**	**Characters**	***Zuphium* (*s. str.*) *ustum***	***Zuphioides mexicanum***	***Coarazuphium whiteheadi***
01	Antennomere 1, row of erect setae	character state 01.1<br/> absent	character state 01.2<br/> present<br/> Fig. 4D	character state 01.2<br/> present<br/> Fig. 4B
02	Posterior supraorbital seta	character state 02.1<br/> absent	character state 02.2<br/> present<br/> Fig. 4C	character state 02.2<br/> present<br/> Fig. 4A
03	Prosternal setae	character state 03.1<br/> absent	character state 03.2<br/> present	character state 03.2<br/> present<br/> Fig. 9B
04	Male adhesive setae	character state 04.1 biseriate	character state 04.2<br/> pad-like,<br/> Fig. 9A	character state 04.2<br/> pad-like
05	Male genitalia, paraostial sclerites	character state 05.1<br/> present<br/> Figs 13A, 13C	character state 05.2<br/> absent	character state 05.2<br/> absent
06	Female genital tract, bursal sac	character state 06.1<br/> absent	character state 06.2<br/> present<br/> Fig. 12A	character state 06.2<br/> present<br/> Fig. 12B
07	Female genital tract, sec. spermathecal gland	character state 07.1<br/> present<br/> Fig. 13D	character state 07.2<br/> absent	character state 07.2<br/> absent
08	Integument color	character state 08.1<br/> rufous to piceous	character state 08.1<br/> rufous	character state 08.2<br/> testaceous
09	Head capsule form	character state 09.1 trapezoidal	character state 09.1 trapezoidal<br/> Fig. 4C	character state 09.2<br/> oviform<br/> Fig. 4A
10	Eyes	character state 10.1 macrophthalmous	character state 10.1 macrophthalmous<br/> Fig. 3B	character state 10.2 microphthalmous<br/> Fig. 3A<br/> or anophthalmous
11	Metepisternum	character state 11.1<br/> elongate	character state 11.1<br/> elongate	character state 11.2<br/> quadrate
12	Hind wings	character state 12.1 macropterous	character state 12.1 macropterous	character state 12.2 brachypterous-apterous
13	Ovipositor: gonocoxite 2	character state 13.1<br/> distal margin evenly rounded	character state 13.1<br/> distal margin evenly rounded<br/> Fig. 11F	character state 13.2<br/> distal margin deeply notched<br/> Fig. 11C

*Zuphioides mexicanum* shares character states 01.2, 02.2, 03.2, 04.2, 05.2, 06.2, and 07.2 with *Coarazuphium whiteheadi*; <br/> *Zuphioides mexicanum* shares character states 08.1, 09.1, 10.1, 11.1, 12.1, and 13.1 with *Zuphium ustum*; <br/> *Zuphium ustum* exhibits uniquely character states 01.1, 02.1, 03.1, 04.1, 05.1, 06.1, and 07.1;<br/> *Coarazuphium whiteheadi* exhibits uniquely character states 08.2, 09.2, 10.2, 11.2, 12.2, and 13.2.

### *Zuphium* genus-group in the Western Hemisphere

Included are two precinctive genera of Zuphiini: *Coarazuphium* Gnaspini et al., and *Zuphioides*, **new genus**. They share the following features.

**Recognition.** With features of Western Hemisphere Zuphiini (see “Recognition”, Appendix I) and head markedly constricted posteriorly, a narrow neck formed by posterior part of occiput and postocciput; maxillary and labial palpi of about equal size and form, terminal palpomeres slightly widened apicad, apical margin obliquely truncate; antennomere 1 with short row of erect setae among the decumbent vestitural setae; prosternum with pair of long erect setae; male fore-tarsomeres 1-3 ventrally with adhesive vestiture of articulo-setae, in form of a broad pad; phallus dorsally without paraostial sclerites; endophallus with various microtrichial patches, without sclerites; ovipositor gonocoxite 2 very broad in dorso-ventral aspect without ensiform setae; female reproductive tract with terminal bursal sac, and without bulbous secondary spermathecal gland.

**Description.** *Size and proportions*. Small, OBL 4.13–4.40 mm; EW 1.36–1.48 mm; A1L/A2-4L 1.03-1.11; HW/PW 1.08.

*Color*. Body various, piceous to testaceous, or in life almost white; appendages slightly paler; vestiture flavous.

**Figure 1.**
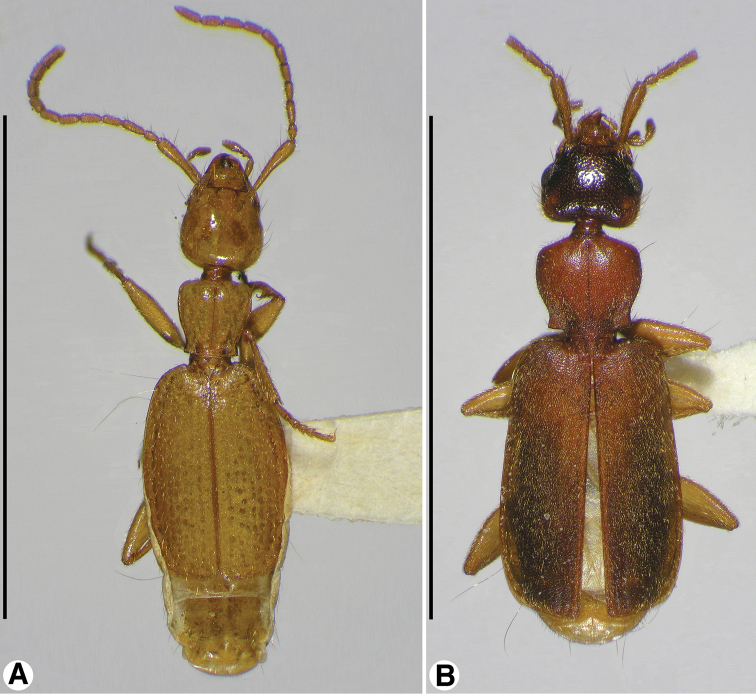
Habitus, dorsal aspect, of: **A** *Coarazuphium whiteheadi*, new species **B** *Zuphioides mexicanum* (Chaudoir). Scale bars = 5 mm.

*Habitus* (Figs 1A, 1B). Flat, overall. Head (dorsal aspect) trapezoidal or oviform, subangulate or broadly rounded posteriolaterally anteriad markedly narrowed neck. Eyes (Figs 3A, 3B) convex, flat, or lacking. Pronotum narrow. Elytra parallel-sided or narrowed both anteriorly and posteriorly, lateral margins distinctly bowed.

*Microsculpture*. Dorsal surface of head (including clypeus) and pronotum smooth, microlines not evident; labrum and elytra with microlines fine (not easily seen at 50× or lower magnification), mesh pattern isodiametric. Ventral surface with microlines fine, generally transverse, sculpticells rather short.

*Luster*. Dorsal surface of head and pronotum shiny, elytra somewhat duller.

*Body vestiture and punctation*. Dorsal surface of head and pronotum sparsely punctate, vestiture sparse. Elytra with punctation and vestiture dense, vestitural setae decumbent.

*Fixed setae*. Head (Figs 4A, 4C): clypeus one pair; head capsule with anterior pair of supraorbitals (**asos**) above eyes; posterior pair of supraorbitals (**psos**) posteriad eyes; one pair of postocular setae (**pos**) immediately behind eyes, laterally; one pair of occipital setae (**ocs**) posteriorly and mediad eyes, or lacking; also one pair posterior supernumerary setae (**psus**) laterad and close to posterior supraorbital setae, or lacking. Antennomere 1 (Figs 4B, 4D, **as1**) with single long seta distally, in addition to decumbent vestiture and row of small erect setae. Pronotum with two pairs of lateral marginal setae, anterior pair in anterior 1/8, posterior pair at posteriolateral angles. Prosternum with one pair setae anterioventrally (Fig. 9B). Elytra: each elytron anteriorly with parascutellar seta; lateral setae about 21 (one group anteriorly, one group posteriorly, and single seta medially. Abdominal sterna IV-VI with or without ambulatory setae, sternum VII with pair of long setae near posterior margin.

**Figure 2.**
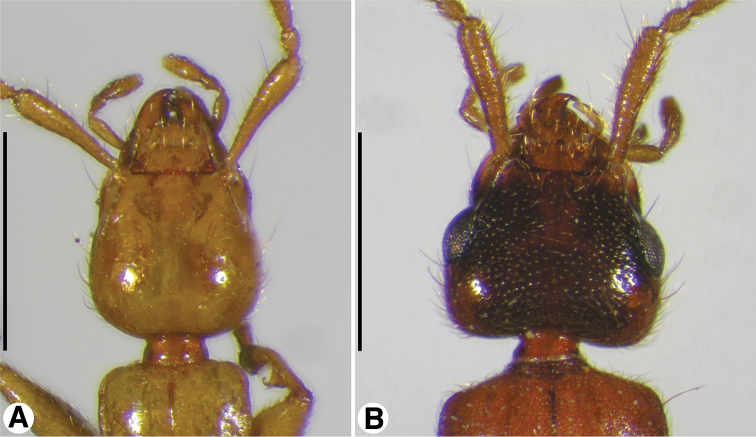
Head, dorsal aspect, of: **A** *Coarazuphium whiteheadi*, new species **B** *Zuphioides mexicanum* (Chaudoir). Scale bars = 1 mm.

*Head*, *dorsal aspect* (Figs 2A, 2B). Occiput posteriorly and postocciput markedly constricted, in form of narrow neck, postoccipital suture evident. Frontoclypeal suture present (most species) or absent, with frons and clypeus fused (*Coarazuphium pains*, Álvares and Ferreira 2002: 41). Genal sulcus prominent.

**Figure 3.**
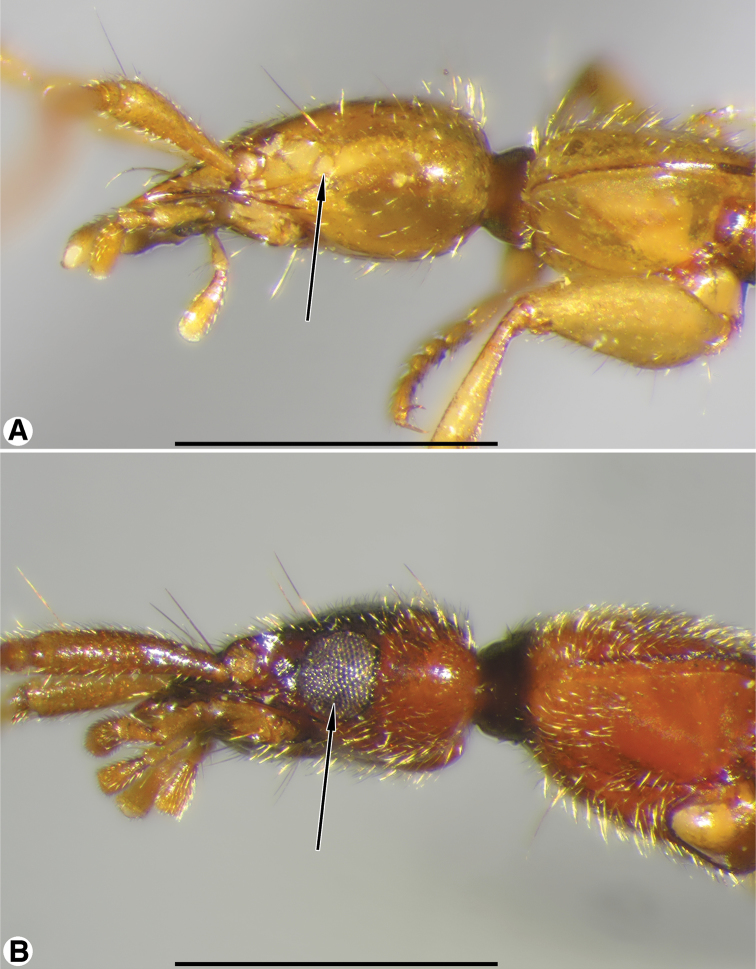
Head and prothorax, lateral aspect, of: **A** *Coarazuphium whiteheadi*, new species **B** *Zuphioides mexicanum* (Chaudoir). Legend: arrow indicates eye. Scale bars = 1 mm.

*Eyes*. Small (microphthalmous), flat (Fig. 3A), ommatidia not evident at 50× or lower magnification; or, eyes normally convex (macrophthalmous, Fig. 3B), ommatidia evident at 50×.

**Figure 4.**
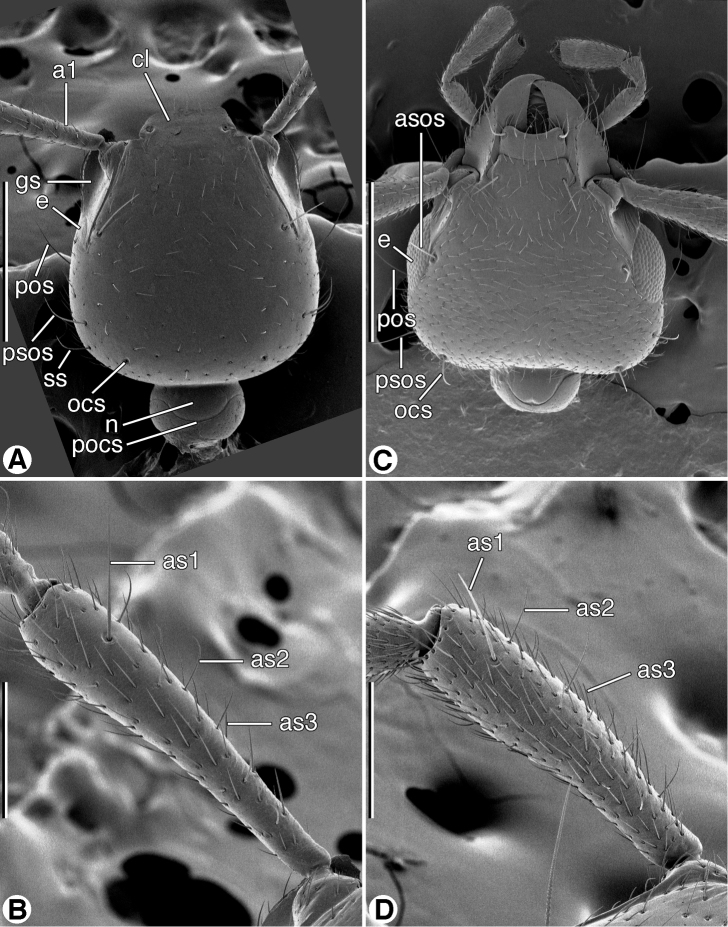
SEM micrographs of head and antennomere 1, dorsal aspect. **A** and **B** *Coarazuphium whiteheadi*, new species **C** and **D** *Zuphioides mexicanum* (Chaudoir). Legend: **a1**, antennomere 1; **as1, as2, as3,** erect setae on antennomere 1; **asos**, anterior supraorbital seta; **cl**, clypeus; **e**, eye; **gs**, genal sulcus; **n**, neck; **ocs**, occipital seta; **pocs**, postoccipital suture; **pos**, postocular seta; **psos**, posterior supraorbital seta; **ss**, posterior supernumerary seta. Scale bars: **A** and **C** = 500 µm; **B** and **D** = 200 µm.

*Antennae* (Fig. 1A). Filiform, extended about body length. Antennomere 1 (Figs 4B, 4D, **a1**) rather slender, slightly longer than antennomeres a2-a4; antennomere 2 short, about half length of a3; antennomeres 3-11 narrow, cylindrical, distinctly longer than wide.

**Figure 5.**
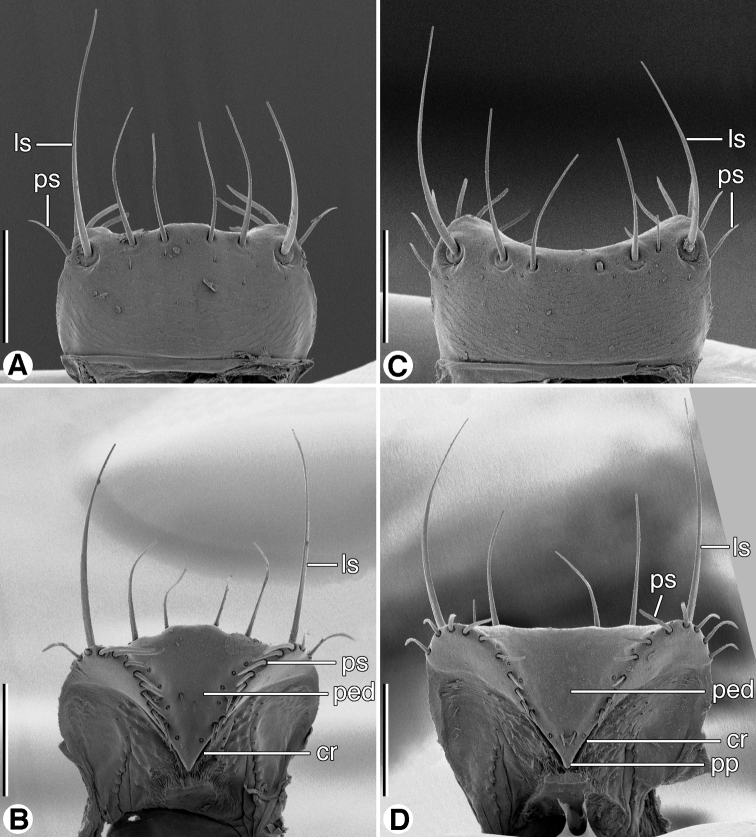
SEM micrographs of labra. **A** and **B** dorsal and ventral aspects, respectively, of *Coarazuphium whiteheadi*, new species **C** and **D** dorsal and ventral aspects, respectively, of *Zuphioides mexicanum* (Chaudoir). Legend: **cr**, crepis; **ls**, lateral seta; **ped**, pedium; **pp**, parapedial projection; **ps**, parapedial seta. Scale bars = 100 µm.

*Labrum* (Figs 5A, 5C). Rectangular, lateral margins rounded; anterior margin concave or irregularly convex; six dorsal setae, lateral setae longer than four medials. Epipharynx (Figs 5B, 5D) with pedium (**ped**) rather broad; parts standard (crepis **cr**, parapedial projection (**pp**), and parapedial setae (**ps**).

**Figure 6.**
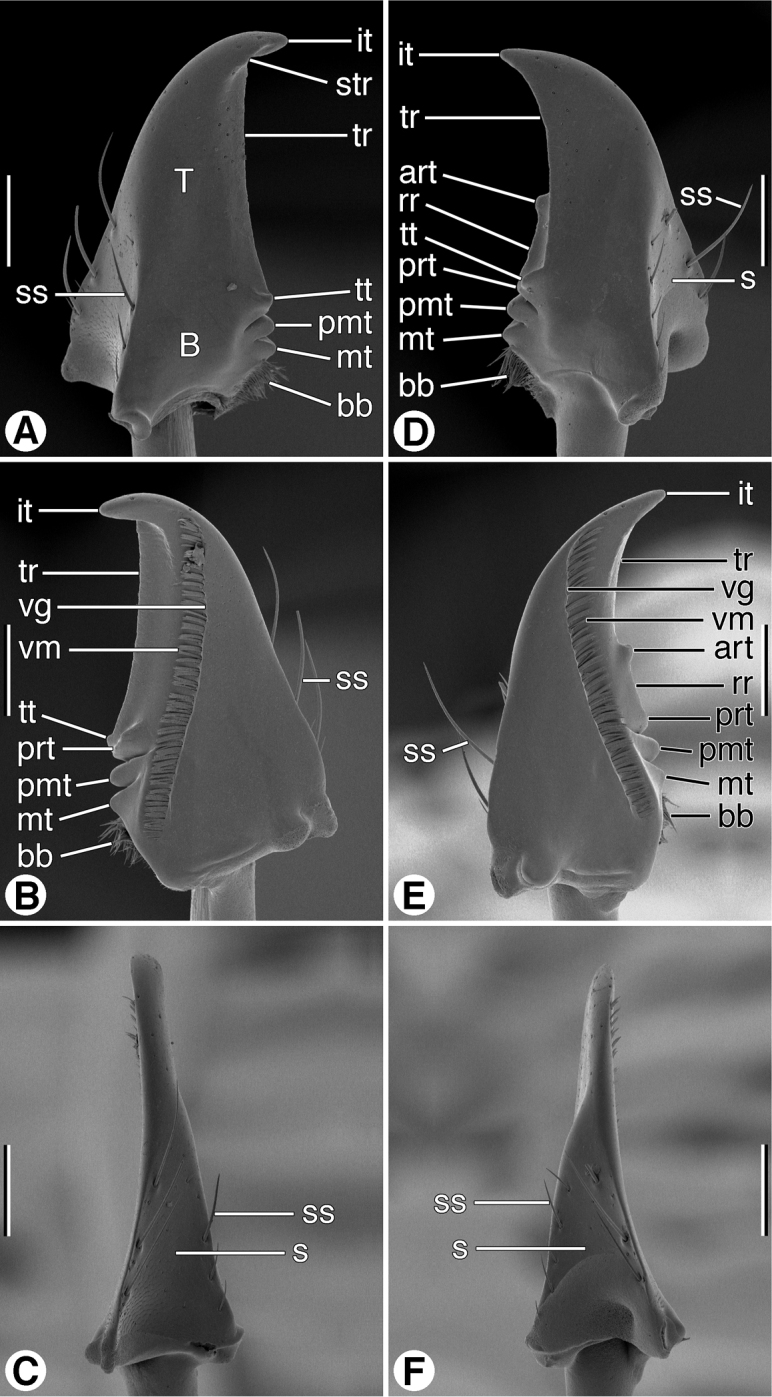
SEM micrographs of mandibles of *Coarazuphium whiteheadi*, new species. **A** and **D** dorsal aspect **B** and **E** ventral aspect **C** and **F** lateral aspect. Legend: **art**, anterior retinacular tooth; **B**, basal area; **bb**, basal brush; **it**, incisor tooth; **mt**, molar tooth; **pmt**, premolar tooth; **prt**, posterior retinacular ridge; **rr**, retinacular ridge; **s**, scrobe; **ss**, scrobal seta; **str**, supraterebral ridge; **T**, terebra; **tr,** terebral ridge; **tt**, terebral tooth; **vg**, ventral groove; **vm**, ventral microtrichia. Scale bars = 100 µm.

**Figure 7.**
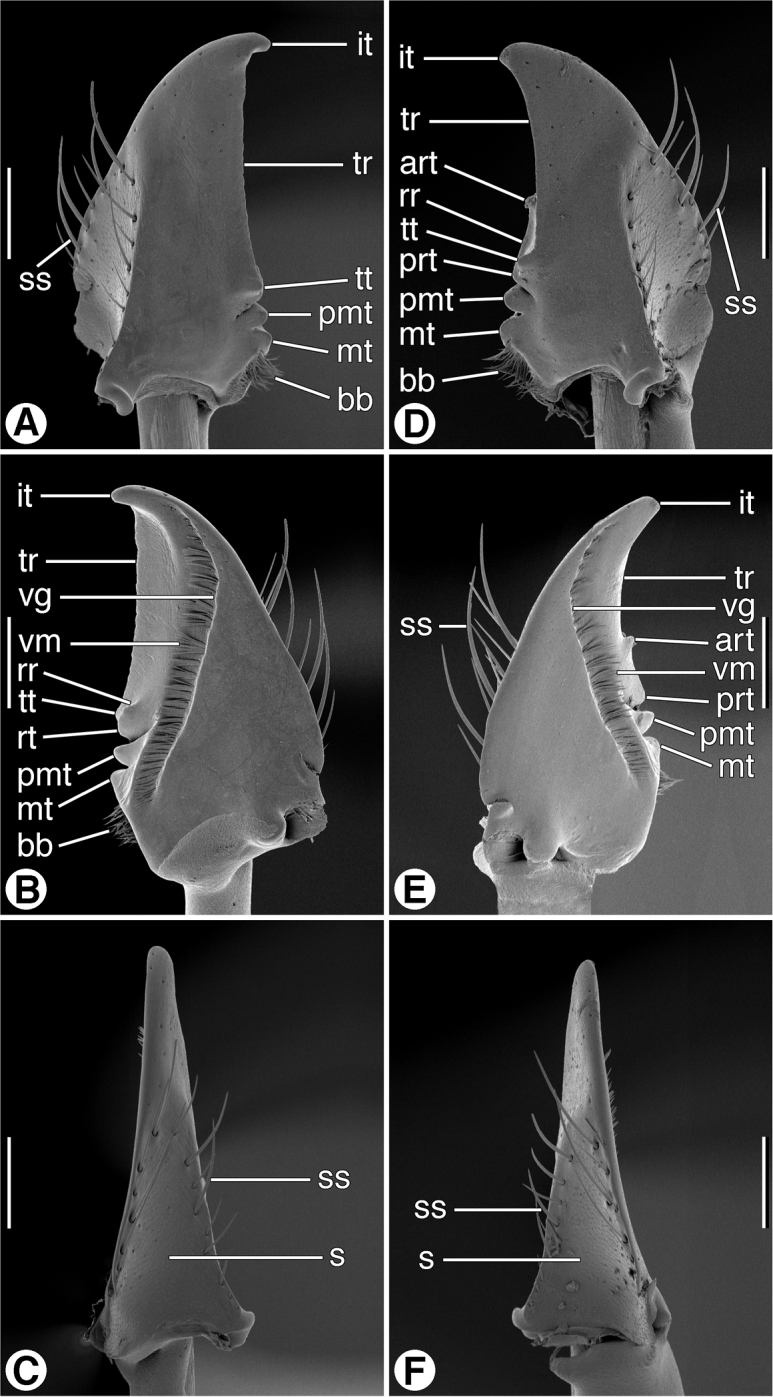
SEM micrographs of mandibles of *Zuphioides mexicanum* (Chaudoir). **A** and **D** dorsal aspect **B** and **E** ventral aspect **C** and **F** lateral aspect. Legend: **art**, anterior retinacular tooth; **bb**, basal brush; **it**, incisor tooth; **mt**, molar tooth; **pmt**, premolar tooth; **prt**, posterior retinacular ridge; **rr**, retinacular ridge; **s**, scrobe; **ss**, scrobal seta; **tr,** terebral ridge; **tt**, terebral tooth; **vg**, ventral groove; **vm**, ventral microtrichia. Scale bars = 100 µm.

*Mandibles* (Figs 6A–6F and 7A–7F). Trigonal in form, with long rather slender terebra (**T**) and short basal area (**B**) with condyles; occlusal margin with long terebral ridge (**tr**), prominent incisor tooth (**it**), blunt terebral tooth (**tt**), small premolar (**pmt**) and molar (**mt**) teeth, and short basal brush (**bb**); ventrally with long ventral groove (**vg**) extended apically almost to mandibular margin, with moderately long and dense microtrichia (**vm**); lateral margin basally with distinct trianguloid scrobe (**s**), dorsal and ventral margins each with three moderately long setae (**ss**). Left mandible in dorsal aspect (Fig. 6A) apically with rather broad incisor tooth (**it**), short supraterebral ridge (**str**), shallowly concave terebral ridge (**tr**), moderately prominent terebral tooth (**tt**), and distinct premolar and molar teeth (**pmt** and **mt**); ventral surface (Figs 6B) with blunt retinacular tooth (**rt**), and short retinacular ridge (**rr**); other features as noted above. Right mandible, dorsal aspect (Figs 7A) with incisor tooth (**it**) shorter and less curved than in left mandible, terebral tooth (**tt**) not prominent; retinaculum prominent, with anterior and posterior teeth (**art** and **prt**, respectively), and intervening retinacular ridge (**rr**); terebral ridge (**tr**); premolar and molar teeth (**pmt** and **mt**, respectively) evident.

**Figure 8.**
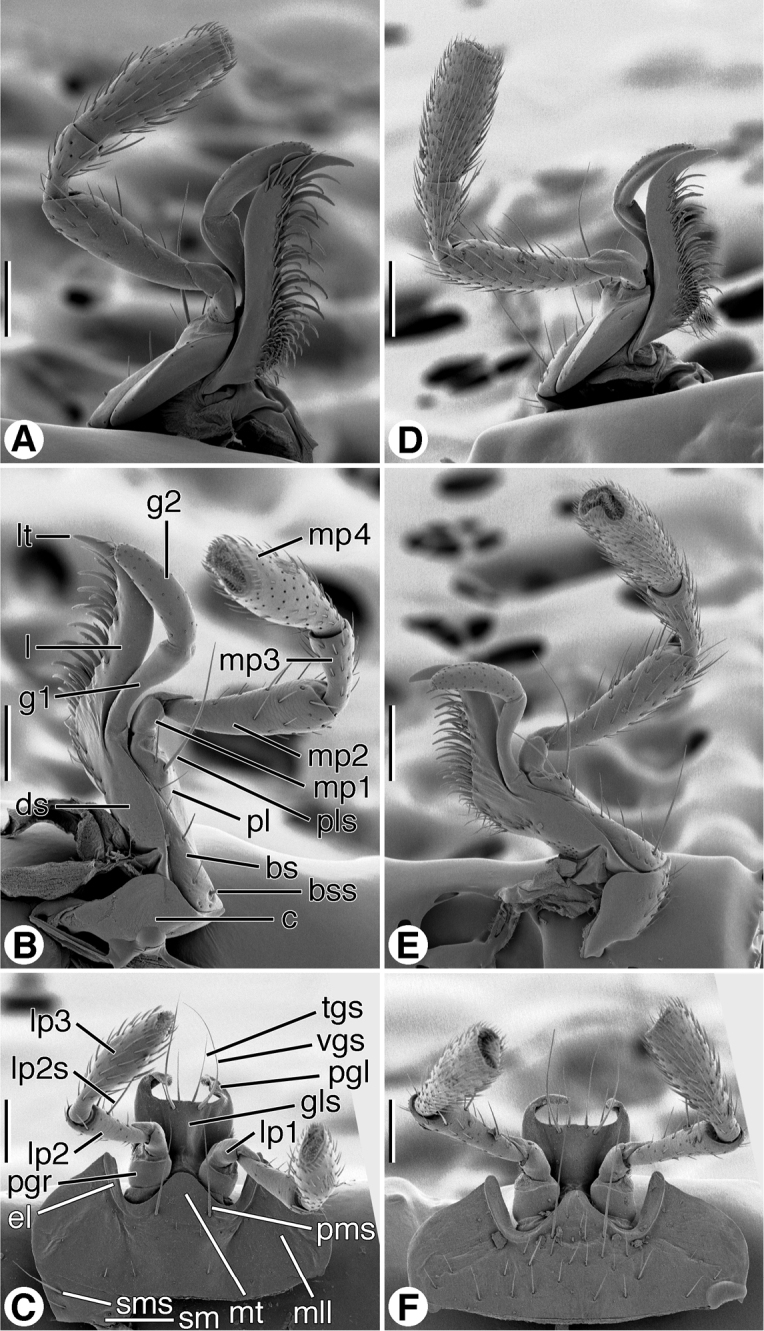
SEM micrographs of left maxillae and labia. **A**–**C** *Coarazuphium whiteheadi*, new species **D**–**F** *Zuphioides mexicanum* (Chaudoir). Legend: **bs**, basistipes; **bss**, basistipital seta; **c**, cardo; **ds**, distitipes; **el**, epilobe; **g1**, galeomere 1; **g2**, galeomere 2; **gls**, glossal sclerite; **l**, lacinia; **lp1**, labial palpomere 1; **lp2**, labial palpomere 2; **lp2s**, labial palpomere 2 seta; **lp3**, labial palpomere 3; **lt**, lacinial tooth; **mll**, mental lateral lobe; **mp1**, maxillary palpomere 1; **mp2**, maxillary palpomere 2; **mp3,** maxillary palpomere 3; **mp4**, maxillary palpomere 4; **mt**, mental tooth; **pgl**, paraglossa; **pgr**, palpiger; **pl**, palpifer; **pls**, palpifer seta; **pms**, paramedial mental seta; **sm**, submentum; **sms**, submental seta; **tgs**, terminal glossal seta; **vgs**, ventral glossal seta. Scale bars = 100 µm.

*Maxillae* (Figs 8A, 8B, 8D, 8E; left maxilla illustrated). Standard sclerites: cardo (**c**), stipes (basistipes (**bs**), dististipes (**ds**) and palpifer (**pl**)), galea (galeomere 1 (**g1**) and galeomere 2 (**g2**)); palpus with four palpomeres (**mp1**-**mp4**). Marginal setae: basistipital (**bss**) and palpiferal (**pls**). Lacinial occlusal margin densely setose, setae thick, moderately long. Galeomere 2 with numerous short erect setae; palpomeres 2 and 3 (**mp2, mp3**) sparsely setose; palpomere 4 (**mp4**) moderately densely setose. Palpomere 4 widened slightly distally, apical margin subtruncate.

**Figure 9.**
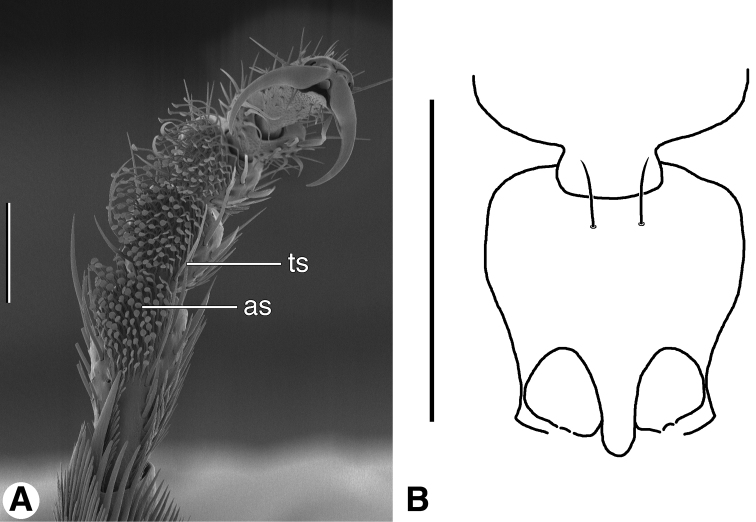
**A** SEM micrograph of fore tarsus of *Zuphioides mexicanum* (Chaudoir). Legend: **as**, articulo-seta; **ts**, trichoid seta. Scale bar = 100 µm. **B** Line drawing of prothorax and base of head, ventral aspect, of *Coarazuphium whiteheadi*, new species. Only medial pair of prominent prosternal setae illustrated, vestiture omitted. Scale bar = 1 mm.

*Labium*, *ventral aspect* (Figs 8C, 8F). Standard sclerites: submentum (**sm**), mentum, and prementum; prementum comprised of a central glossal sclerite (**gls**), pair of lateral paraglossae (**pgl**), and pair of palpi, each of three articles (**lp1**- **lp3**), borne on a short broad palpiger (**pgr**). Submentum (**sm**) narrow rectangular sclerite, with pair of lateral setae (**sms**). Mentum transverse, with broad lateral lobes (**mll**), broad, blunt tooth (**mt**), broad epilobes (**el**); one pair of long paramedial setae (**pms**). Glossal sclerite (**gls**), fused laterally each side with basal part of narrow paraglossae (**pgl**), latter membranous, densely setose, extended distally beyond broad distal margin of glossal sclerite. Glossal sclerite with pair of longer ventral setae (**vgs**), and four shorter terminal marginal setae (**tgs**). Palpigers (**pgr**) cup-like, glabrous. Palpi each of three palpomeres (**lp1**- **lp3**), palpomere 1 short, glabrous; palpomere 2 long, slender, subcylindrical, and moderately densely setose, most setae erect, one pair on anterior margin (**lp2s**) long; palpomere 3 widened slightly distally, apical margin subtruncate.

*Pronotum* (Figs 1A, 1B). Anterior margin truncate, lateral margins markedly sinuate posteriorly, posteriolateral angles prominent, dentiform, slightly anteriad posterior margin; surface impressions (anterior and posterior transverse and median longitudinal) shallow; lateral grooves and posteriolateral impressions moderately deep.

*Pterothorax*. Metasternum moderately long or short; metepisternum longer than wide or quadrate.

*Elytra* (Figs 1A, 1B). Separate, not fused along suture. Each elytron more or less rectangular, lateral margin straight or bowed, humerus projected anteriorly, basal ridge sinuate; apical margin truncate, with narrow band of membrane. surface with striae very shallow, intervals almost flat.

*Hind wings*. Macropterous, or brachypterous, with each wing represented by short stub.

*Abdomen*. Sternum VII with apical margin truncate.

**Figure 10.**
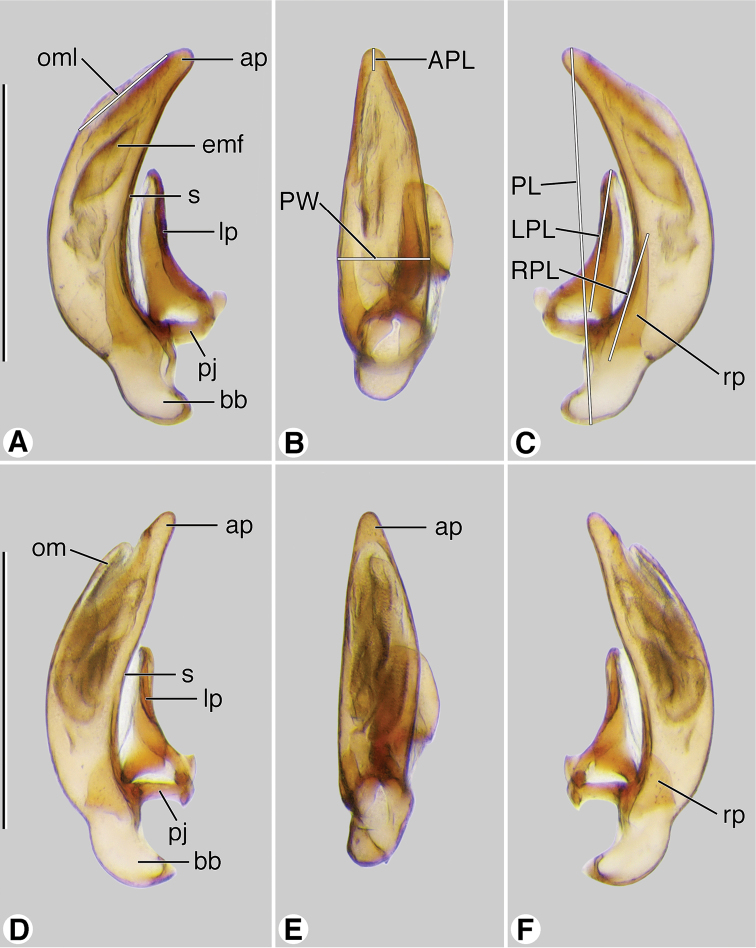
Digital images of male genitalia. **A**–**C** *Coarazuphium whiteheadi*, new species **D**–**F** *Zuphioides mexicanum* (Chaudoir). **A**, **D** left lateral aspect **B**, **E** dorsal aspect **C**, **F** right lateral aspect. Legend: **ap**, apical portion of phallus; **APL** length of apical portion **; bb**, basal bulb of phallus; **emf**, endophallic terminal microtrichial field; **lp**, left paramere; **LPL**, left paramere length; **om**, ostial membrane; **OML**, periostial membrane length; **pj**, parameral juxta; **PL**, phallus length; **PW**, phallus width; **rp**, right paramere; **RPL**, right paramere length; **s,** shaft of phallus. Scale bars = 0.5 mm.

*Male genitalia* (Figs 10A–10F). Anopic. Phallus slightly curved ventrally, narrowed apically, apical margin rounded, apical portion very short; without paraostial sclerites. Left paramere (**lp**) conchoid or styliform. Right paramere (**rp**) short, broad, or styliform, relatively long.

**Figure 11.**
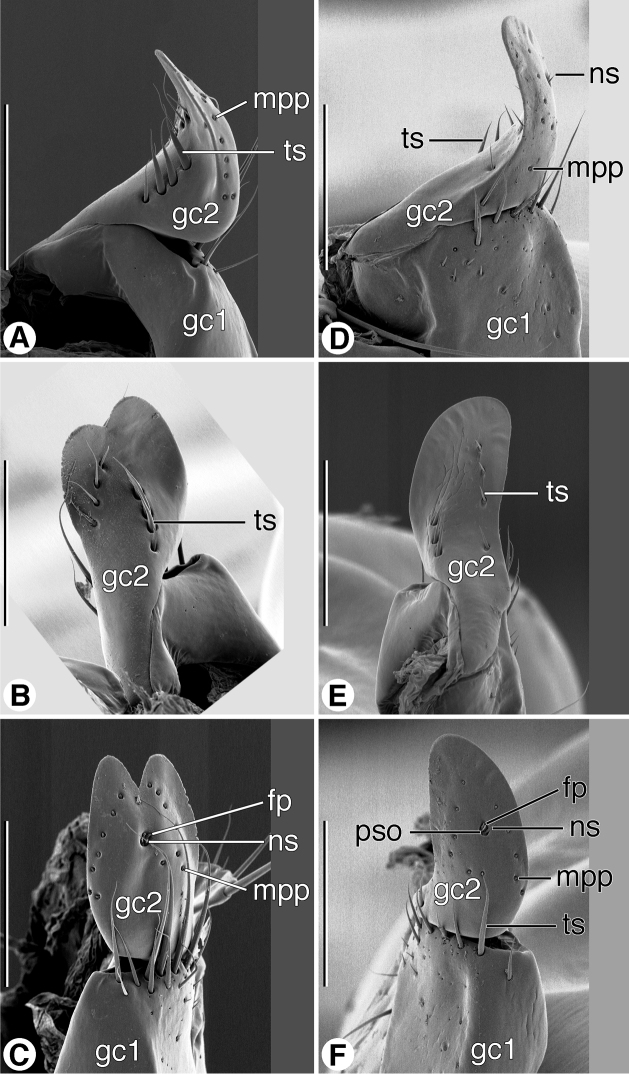
SEM micrographs of female ovipositor sclerites. **A**–**C** *Coarazuphium whiteheadi*, new species **D**–**F** *Zuphioides mexicanum* (Chaudoir). **A**, **D** left lateral aspect **B**, **E** dorsal aspect **C**, **F** ventral aspect. Legend: **fp**, furrow peg; **gc1**, gonocoxite 1; **gc2**, gonocoxite 2; **mpp**, marginal pit pegs; **ns**, nematiform seta; **pso**, preapical setose organ; **ts**, trichoid seta. Scale bars = 100 µm.

*Female genitalia*: *ovipositor* (Figs 11A–11F). Gonocoxite 1 (**gc1**) short, thick, with or without row of long trichoid setae distally on ventral and lateral surface. Gonocoxite 2 (**gc2**) short, thick; in lateral aspect falciform, apex pointed, variously provided with long trichoid setae dorso- and ventrolaterally; in dorsoventral aspect, broad, paddle-like, apex deeply notched or not; dorsal surface glabrous except for dorsolateral and ventrolateral trichoid setae (**ts**); ventral surface toward margins with row of pit pegs (**mpp**), preapical setose organ (**pso**) circuloid, with two furrow pegs (**fp**) and two very short nematiform setae (**ns**).

**Figure 12.**
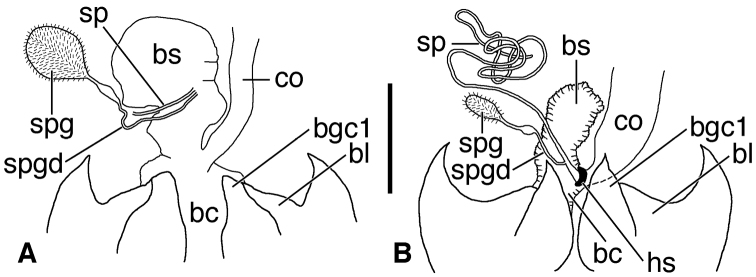
Line drawings of female reproductive tract, ventral aspect. **A** *Coarazuphium whiteheadi*, new species **B** *Zuphioides mexicanum* (Chaudoir). Legend: **bc**, bursa copulatrix; **bl**, base of laterotergite; **bs**, bursal sacculus; **bgc1**, base of gonocoxite 1; **co**, common oviduct; **hs**, helminthoid sclerite; **sp**, spermatheca; **spg**, spermathecal gland; **spgd**, spermathecal gland duct. Scale bar = 0.25 mm.

*Female genital tract* (Figs 12A, 12B). Bursa copulatrix (**bc**) ended in an expanded bulbous anterior extension (**bs**). Common oviduct (**co**) inserted in bursa copulatrix at base of its anterior extension. Spermatheca (**sp**) slender, long, inserted on or at base of bursal sac, beside insertion point of spermathecal gland duct (**spgd**); latter with swelling proximad spermathecal gland (**spg**). With or without helminthoid sclerite. Without secondary spermathecal gland.

**Figure 13.**
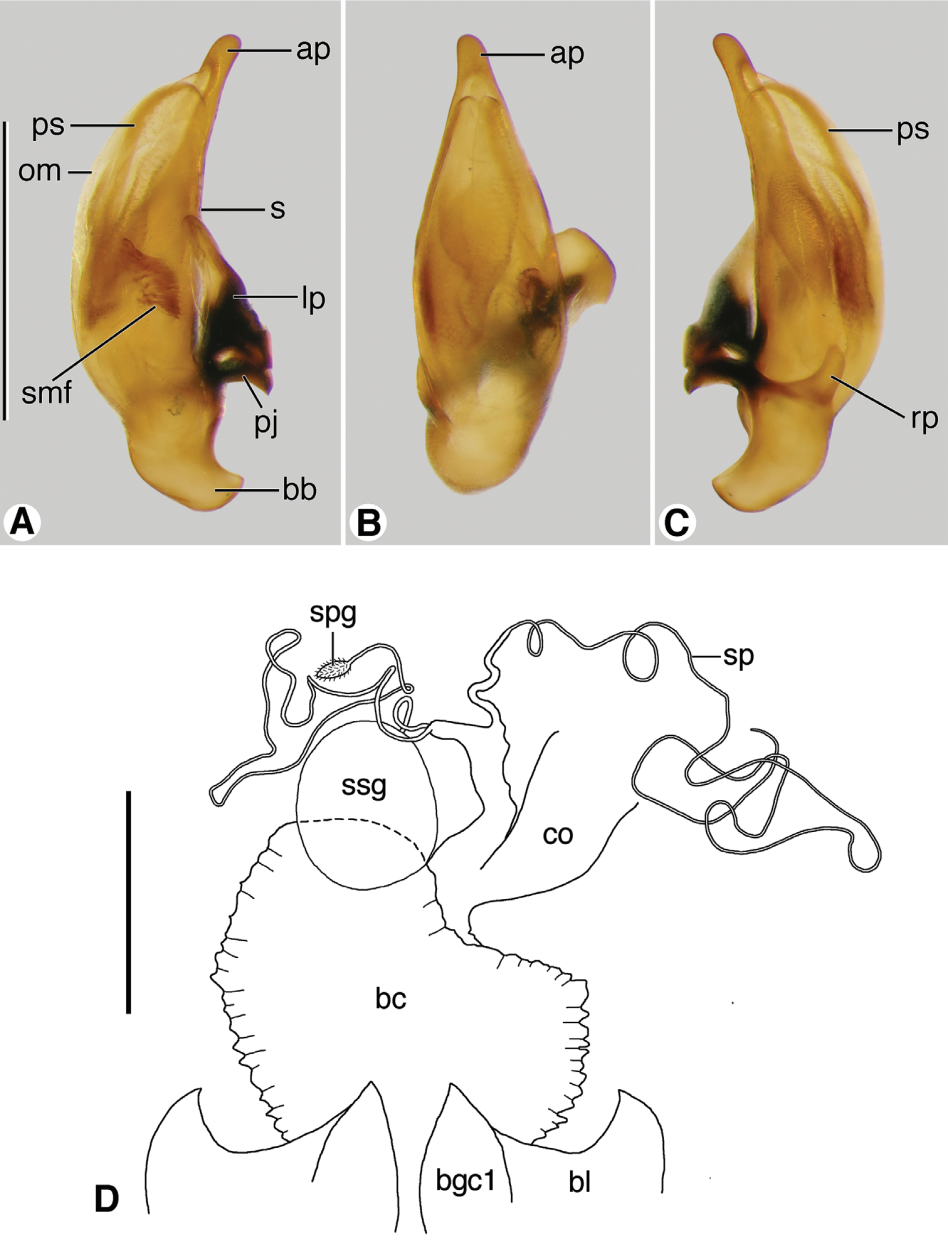
Digital images of male genitalia and line drawing of female reproductive tract of *Zuphium ustum* Klug. **A**–**C** male genitalia, left lateral, dorsal and right lateral aspects, respectively **D** female reproductive tract, ventral aspect. Legend: **ap**, apical portion of phallus; **bb**, basal bulb of phallus; **bc,** bursa copulatrix; **bl**, base of laterotergite; **bgc1**, base of gonocoxite 1; **co**, common oviduct; **lp**, left paramere; **rp**, right paramere; **om**, ostial membrane; **pj**, parameral juxta; **ps**, paraostial sclerites; **rp**, right paramere; **s**, shaft of phallus; **smf**, spiny microtrichial field; **sp**, spermatheca; **spg**, spermathecal gland; **ssg**, secondary spermathecal gland. Scale bars: **A** – **C** = 1 mm; **D** = 0.5 mm.

**Geographical distribution.** The range of this genus-group in the Western Hemisphere extends from Neotropical Argentina (Reichardt 1977: 449) northward through the American tropics and subtropics to temperate eastern and central U.S.A., and to southeastern Canada (Lindroth 1969: 1090), with an isolated population of *Zuphioides americanum* in western Oregon (Bousquet 2012: 1356).

**Included taxa.** The *Zuphium* genus-group in the Western Hemisphere includes 30 species arranged in two genera: *Coarazuphium* Gnaspini et al. and *Zuphioides* new genus (Table 2).

**Table 2.** List of names of species of *Coarazuphium* Gnaspini, Vanin & Godoy and *Zuphioides*, new genus.<br/>

*Coarazuphium bezerra* Gnaspini, Vanin & Godoy 1998
*Coarazuphium cessaima* Gnaspini, Vanin & Godoy 1998
*Coarazuphium formoso* Pellegrini & Ferreira 2011
*Coarazuphium pains* Alvarez & Ferreiera 2002
*Coarazuphium tapiaguassu* Pellegrini & Ferreira 2011
*Coarazuphium tessai* (Godoy & Vanin) 1990
*Coarazuphium whiteheadi* Ball & Shpeley, sp. n.
*Zuphioides aequinoctiale* (Chaudoir, 1862)
*Zuphioides americanum* (Dejean, 1831)
*Zuphioides argentinicum* (Liebke, 1933)
*Zuphioides batesi* (Chaudoir, 1862)
*Zuphioides bierigi* (Liebke, 1933)
*Zuphioides brasiliense* (Chaudoir, 1872)
*Zuphioides bruchi* (Liebke, 1933)
*Zuphioides capitum* (Liebke, 1933)
*Zuphioides columbianum* (Chaudoir, 1872)
*Zuphioides cubanum* (Liebke, 1933)
*Zuphioides delectum* (Liebke, 1933)
*Zuphioides exiguum* (Putzeys, 1878)
*Zuphioides exquisitum* (Liebke, 1933)
*Zuphioides flohri* (Liebke, 1933)
*Zuphioides haitianum* (Darlington, 1935)
*Zuphioides lizeri* (Liebke, 1933)
*Zuphioides longicolle* (LeConte, 1879)
*Zuphioides magnum* (Schaeffer, 1910)
*Zuphioides mexicanum* (Chaudoir, 1863)
*Zuphium vivinum* (Liebke, 1933)
*Zuphioides pseudamericanum* (Mateu, 1981)
*Zuphioides punctipenne* (Bates, 1891)
*Zuphioides pusillum* (Chaudoir, 1862)
*Zuphioides salivanum* (Liebke, 1933)

#### Coarazuphium

Gnaspini,Vanin & Godoy, 1998

http://species-id.net/wiki/Coarazuphium

Parazuphium ; Godoy and Vanin 1990 : 795 (not Jeannel 1942).
Zuphium ; Mateu 1993 : 486 (not Latreille 1805).
Coarazuphium Gnaspini, Vanin & Godoy, 1998: 298. Álvares and Ferreira 2002 : 41. Lorenz 2005: 507. Pellegrini and Ferreira 2011a : 39. Pellegrini and Ferreira 2011b: 47.

##### Type species.

*Parazuphium tessai* Godoy & Vanin, 1990 (designated by Gnaspini et al. 1998).

##### Generic name.

As explained by Gnaspini et al. (1998: 299), the name *Coarazuphium* is a compound Latinized neuter noun derived from “coara”, meaning hole or cave in the Tupi language (Brazilian, native tongue), plus *Zuphium*. The name refers to the troglophilic or troglobitic habits of the species of this genus, and their basic nominotypical *Zuphium*-like features.

##### Recognition.

With character states of Western Hemisphere *Zuphium* genus-group, restrictedas follows: size small; pronotum and dorsal surface of elytra moderately densely setose, setae decumbent; body markedly depressed; integument pale; head narrow, oviform, with posteriolateral margins broadly rounded; antennae elongate, antennomere 1 as long or longer than antennomeres 2-4; humeri narrowed, slightly or markedly so; metasternum short, metepisternum quadrate. Brachypterous or apterous. Male genitalia (Figs 10A–10C): phallus without dorsal paraostial sclerites. Female genital tract (Fig. 12A, 12B): without secondary spermathecal gland.

##### Description.

None required here. See Gnaspini et al. (1998: 298-299), Pellegrini and Ferreira (2011b), and description of *Coarazuphium whiteheadi* below.

##### Geographical distribution.

Confined to the Neotropical Region, the seven species of this genus are known only from southeastern Brazil (Pellegrini and Ferreria 2011b: 57, fig. 10) and southern Mexico (Map 1). This is an example of the “Paleo-American distribution pattern” (see Halffter (1987) and Liebherr (1994: 845)).

##### Way of life.

The previously described species of genus *Coarazuphium* inhabit caves in one of two types of substrate (Pellegrini and Ferreira 2011b: 55): limestone, occupied by *Coarazuphium bezerra* Gnaspini, Vanin & Godoy; *Coarazuphium cessaima* Gnaspini, Vanin & Godoy; *Coarazuphium formoso* Pellegrini & Ferreira; *Coarazuphium pains* Álvares & Ferreira; and *Coarazuphium tessai* (Godoy & Vanin); and iron ore, occupied by *Coarazuphium tapiaguassu* Pellegrini & Ferreira. The caves in iron ore are described as “shallow”. Members of those species occupying the caves in limestone substrate are described as freely walking over the soil, and presumably resting exposed, rather than resting (hiding?) under rock cover. In contrast, all of the specimens collected of *Coarazuphium tapiaguassu* were found under rocks, on the cave floor. In contrast, known members of the Mexican species, *Coarazuphium whiteheadi*, new species, seem to be surface inhabitants (see below)

##### Parasites.

Some specimens of *Coarazuphium tapiaguassu* were infested by laboulbenelian fungi. The fungi were not identified further. See this topic for *Coarazuphium whiteheadi*, below.

##### Evolutionary considerations.

The ultrastructural features (i.e., principally sensillar) observed with scanning electron microscopy by Pellegrini and Ferreria (2011a, 2011b) differ only slightly between the two species of *Coarazuphium* that they studied, and between that genus and *Zuphioides* (cf. accompanying SEM illustrations of *Zuphioides mexicanum*). So, it seems to us unlikely that such features are or will be evolutionarily informative.

In contrast, the standard structural troglobitic features of *Coarazuphium* (lengthening of antennae and legs, depigmentation, micro- or anophthalmy, and reduction of elytral length, elytral humeri, metathorax and metathoracic wings) plus details of male and female genitalia are evolutionarily informative. Based on eye loss and more elongate appendages, Gnaspini et al. (1998: 303) proposed that *Coarazuphium cessaima* showed the more modified features, compared to *Coarazuphium tessai* and *Coarazuphium bezerra*, the only other species of *Coarazuphium* known at that time. Álvares and Ferreira (2002: 43) proposed that, based on the features noted above, their newly described *Coarazuphium pains* would occupy a position intermediate between *Coarazuphium cessaima* and *Coarazuphium tessai* + *Coarazuphium bezerra*.

The external features of *Coarazuphium* evidently evolved in parallel with, and independent of, four other zuphiine troglobite taxa: the remarkable Spanish *Ildobates neboti* Español, 1966; the Canary Islands *Parazuphium feloi* Machado (1997: 163); the Australian Nullarbor *Speozuphium poulteri* Moore (1995: 159) and *Speothalpius grayi* Moore (1995: 160). Another Canary Islands *Parazuphium* (*Parazuphium damascenum canariense* Machado, 1992: 580) exhibits these same reductive (though less developed) features, but it is evidently conspecific with the continental Palaearctic *Parazuphium damascenum damascenum* (Fairmaire, 1897). For a more detailed discussion of the matter see Machado (1992: 581–582).

Gnaspini et al. (1998: 308–309) proposed for the Brazilian species of *Coarazuphium* that “..... these highly derived troglobitic features are due to a long-term isolation inside the subterranean environments, which took place under the drier climate to which the region was in the past and is still submitted in the present. It is largely accepted in the literature that cave arthropods are related to litter epigean and/or endogean ancestors, which already inhabited humid habitats. Therefore,... ... the ancestral species [of *Coarazuphium*] should have been epigean and lived in forested (or at least humid) areas, and occurred at least in part of the region where the genus occurs nowadays. From [such ancestral stock] several lineages invaded the caves from the northern Bambui Speleological Province, where they became isolated with the progressive shrinkage of humid environments. Thence, the origin of the genus takes back to a time when the area was not drying yet, which is probably the Tertiary”.

Discovery of the Mexican species adds important details to the story of evolution of *Coarazuphium*. First, the marked geographical range extension (from southeastern South America to the southern part of the North American continent) shows that this genus was not confined to eastern Brazil. Further, the distribution pattern lends support to the hypothesis of Gnaspini et. al. (see above) that *Coarazuphium* originated in early Tertiary time (i.e., before the beginning of the drying trend that extended through much of the Cenozoic era). Second, the extra-speleal humid forest existence of *Coarazuphium whiteheadi* suggests that the basic troglobitic features of *Coarazuphium* (microphthalmy, brachyptery, depigmentation, etc.) evolved in surface habitats, though probably in forested deep leaf litter locations, and were in effect preadaptations for cave life.

An additional observation relating to evolutionary history of the *Zuphium* genus groupis that shared features of *Zuphioides* and *Coarazuphium* indicate that these two genera may be adelphotaxa, with *Zuphioides* retaining mostly ancestral features including life in lowland hygrophilous or mesophilous situations.

**Key to species of genus *Coarazuphium* Gnaspini, Vanin & Godoy**

**Table:** 

1	Anophthalmous (Gnaspini et al. 1998: 307, fig. 6). Maximum width of elytra near middle. Male genitalia: right paramere (Gnaspini et al. 1998: 306, fig. 10) styliform, about as long as left paramere	*Coarazuphium cessaima* Gnaspini, Vanin & Godoy
1’	Microphthalmous. Maximum width of elytra near middle, or posteriad middle. Male genitalia: right paramere styliform or not, distinctly shorter than left paramere	2
2(1’)	Elytron with apical margin truncate, not sinuate. Male right paramere styliform or broad	3
2’	Elytron with apical margin sinuate. Male right paramere broad, not styliform, distinctly shorter than left paramere	5
3(2)	Head dorsally without setae posteriad the anterior supraorbital setae (Pellegrini and Ferreira 2011: 49, fig. 2A)	*Coarazuphium tapiaguassu* Pellegrini & Ferreira
3’	Head dorsally with one to three pairs of setae posteriad the anterior supraorbital setae	4
4(3)	Labrum with anterior margin broadly concave. Prosternal setae two pair (Álvares and Ferreira 2002: 42, fig. 3). Maximum width (of elytra) posteriad transverse midline. Male right paramere (Álvares and Ferreira 2002: 42, fig. 6) broad, not styliform, distinctly shorter than left paramere. Brazil	*Coarazuphium pains* Álvares & Ferreira
4’	Labrum (Fig. 5A) with anterior margin irregularly convex. Prosternal setae one pair (Fig. 9B). Male right paramere (Fig. 10C, rp) styliform, more than half length of left paramere. Mexico	*Coarazuphium whiteheadi*, sp. n.
5(2’)	Head dorsally with three pairs of setae posteriad the anterior supraorbital setae (Pellegrini and Ferreira 2011a: figs 1A, 1B)	*Coarazuphium formoso* Pellegrini & Ferreira
5’	Head dorsally with one or two pairs of setae posteriad the anterior supraorbital setae	6
6(5’)	Head dorsally with two pairs of setae (posterior supraorbitals and occipitals,) at posterior border of head capsule (Gnaspini et al. 1998: 304, fig. 1). Male left paramere broad, conchoid (Gnaspini et al. 1998: 305, fig. 3)	*Coarazuphium bezerra* Gnaspini, Vanin & Godoy
6’	Single pair of setae (posterior supraorbitals) at posterior border of head capsule (Godoy and Vanin 1990: 796, fig. 1). Male left paramere styliform (Godoy and Vanin 1990: 798, fig. 2)	*Coarazuphium tessai* (Godoy & Vanin)

### Species treatment

Only the following new species is treated here.

#### Coarazuphium whiteheadi sp. n.

urn:lsid:zoobank.org:act:1CC227B6-FA52-40DC-8FC4-C25A060CE300

http://species-id.net/wiki/Coarazuphium_whiteheadi

Figs 1A , 2A , 3A , 4A, 4C , 5A, 5B , 6A–6F , 8A–8C , 9B , 10A–10C , 11A–11C , 12A , Map 1

##### Type material.

Three specimens, as follows. HOLOTYPE female, labeled: “MEXICO, Oaxaca/ 7100’ 21.8 mi./ n. Juchatengo/ VII.18-19.1966”; “George E. Ball/ D. R. Whitehead/ collectors” (USNM). PARATYPES two: female, labeled same as holotype (UASM); male, labeled: “MEXICO, Oaxaca/ Mt. Alban, near ruins/ Acacia scrub 6000 ft/ VI-3/4-82/ Rolf L. Aalbu, col.” (USNM).

##### Type locality.

Ridge top, Sierra de Miahuatlan, in western Oaxaca, Mexico, at 2164 m, 35 km north of San Pedro Juchatengo, 16.462N, 97.010W.

##### Specific epithet.

A Latinized eponym, masculine gender, genitive case, based on the surname of Donald R. Whitehead, now deceased, one of the collectors of the type series of this species.

##### Recognition.

See key, above.

##### Description.

*Size and proportions*. Small, OBL 4.13–4.40 mm; EW 1.36–1.48 mm A1L/A2-4L 1.03–1.11; HW/PW 1.08.

*Color*. Body testaceous, in life almost white; appendages slightly paler; vestiture golden.

*Habitus* (Fig. 1A). Flat, overall. Head capsule (dorsal aspect, Fig. 2A) oviform, broadly rounded posteriolaterally. Eyes (Fig. 3A) flat, hardly perceptible, ommatidia not evident at 50×. Pronotum narrow. Elytra narrowed anteriorly and posteriorly, lateral margins distinctly bowed.

*Microsculpture*. Dorsal surface of head capsule (including clypeus) and pronotum smooth, microlines not evident; labrum and elytra with microlines fine (not easily seen at 50× or lower magnification), mesh pattern isodiametric. Ventral surface with microlines fine, generally transverse, sculpticells rather short.

*Luster*. Dorsal surface of head and pronotum shiny, elytra somewhat duller.

*Body vestiture and punctation*. Dorsal surface of head and pronotum sparsely punctate, vestiture sparse. Elytra with punctation and vestiture dense, vestitural setae decumbent. *Abdomen*. Abdominal sterna IV-VI with punctation rather sparse, vestitural setae decumbent.

*Fixed setae*. Head (Fig. 4A, 4C): clypeus one pair; head capsule with anterior pair of supraorbitals (**asos**) above eyes; posterior pair of supraorbitals (**psos**) posteriad eyes; one pair of postocular setae (**pos**) immediately behind eyes, laterally; one pair of occipital setae (**ocs**) posteriorly and mediad eyes; also one pair posterior supernumerary setae (**psus**) laterad and close to posterior supraorbital setae. Antennomere 1 (Fig. 4B, **as1**) with single long seta distally, and row of several semi-erect setae more proximally (Fig. 4B, **as2**, **as3**). Pronotum with two pairs of lateral marginal setae, anterior pair in anterior 1/8, posterior pair at posteriolateral angles. Prosternum with one pair paramedial setae anterioventrally (cf. Fig. 9B). Elytra: each elytron anteriorly with parascutellar seta; lateral setae about 21 (one group anteriorly, one group posteriorly, and single seta medially). Abdominal sterna IV–VI each with one pair of ambulatory setae, sternum VII with one pair of long setae near posterior margin.

*Head*, *dorsal aspect* (Fig. 1A). Postoccipital suture evident. Frontoclypeal suture present, with frons and clypeus separate. Genal sulcus (Fig. 4A, **gs**) broad, ventral margin sinuous.

*Eyes*. (Fig. 3A) Small, flat, ommatidia not evident at 50×, or lower magnification.

*Antennae*. (Fig. 4A). Filiform, extended about body length. Antennomere 1 (**a1**) rather slender, slightly longer than antennomeres **a2**-**a4**; antennomere 2 short, about half length of **a3**; antennomeres 3–11 narrow, cylindrical, distinctly longer than wide.

*Mouthparts*. As described for *Zuphium* genus-group, above. *Labrum* (Fig. 5A).

Rectangular, lateral margins rounded; anterior margin irregularly convex; six dorsal setae, lateral setae longer than four medials. Epipharynx (Fig. 5B).

*Mandibles* (Figs 6A–6F) *Maxillae* (Figs 8A–8B). *Labium*, *ventral aspect* (Fig. 8C).

*Pronotum* (Fig. 1A). Anterior margin truncate, lateral margins markedly sinuate posteriorly, posteriolateral angles prominent, dentiform, slightly anteriad posterior margin; surface impressions (anterior and posterior transverse and median longitudinal) shallow; lateral grooves and posteriolateral impressions moderately deep.

*Pterothorax*. Metasternum short; metepisternum quadrate.

*Elytra* (Fig. 1A). Separate, not fused along suture. Each elytron more or less rectangular, but narrowed anteriorly and posteriorly, lateral margin thus distinctly bowed; humerus projected anteriorly, basal ridge sinuate; apical margin truncate, with narrow band of membrane; surface with striae very shallow, intervals almost flat.

*Hind wings*. Short stubs, brachypterous.

*Abdomen*. Sternum VII with apical margin truncate.

*Male genitalia* (Figs 10A–10C). Anopic. Phallus narrow (PW/PL 0.238), slightly curved ventrally, narrowed apically, apical margin rounded, apical portion very short (AL/PL 0.063), ostial membrane extensive (OM/PL 0.317). Left paramere (**lp**) conchoid. Right paramere (**rp**) styliform, relatively long (RP/LP 0.317).

*Female genitalia*: *ovipositor* (Figs 11A–11C). Gonocoxite 1 (**gc1**) with patch of long trichoid setae distally on ventral surface. Gonocoxite 2 (**gc2**) short, thick; in lateral aspect falciform, apex pointed, row of four long trichoid setae dorso- and ventro-laterally; in dorso-ventral aspect, broad, paddle-like, apex deeply notched; dorsal surface glabrous except for dorso-lateral and ventro-lateral trichoid setae; ventral surface toward margins with row of pit pegs (**mpp**), preapical setose organ (**pso**) circuloid, with two furrow pegs (**fp**) and two very short nematiform setae (**ns**).

*Female genital tract* (Fig. 12A). Bursa copulatrix (**bc**) ended in an expanded bulbous anterior extension (**bs**). Common oviduct (**co**) inserted in bursa copulatrix at base of the anterior extension. Spermatheca (**sp,** broken) slender, inserted on bursal sac beside insertion point of spermathecal gland duct (**spgd**); latter with swelling proximad spermathecal gland (**spg**). Without helminthoid sclerite. Without secondary spermathecal gland.

##### Collecting notes and habitat.

The specimens from the type locality were collected in the remains of a cloud forest. One was on the ground, in leaf litter; using a Bowie knife, the other was dug out of wet wood of a pine log, near the ground surface. The Monte Alban specimen was likely taken from a dark crevice, “where the temperature was much cooler than outside“ (Rolf Aalbu, personal communication). Judging from the structural features of the type material, this species may be troglophilic, but not troglobitic, as are its Brazilian congeners (Gnaspini et al. 1998; Pellegrini and Ferreira 2011b).

##### Parasites.

Attached to the holotype was a fungus of the species *Rhachomyces zuphii* Thaxter (Laboulbeniales: Laboulbeniaceae) (determiner of fungus not indicated on label), which is now attached to a pinned rectangular piece of clear plastic by a drop of balsam.

##### Geographical distribution

(Map 1). This species is known only from two montane localities in the Mexican state of Oaxaca.

##### Material examined.

Type specimens, only. For label details, see above.

#### Zuphioides gen. n.

urn:lsid:zoobank.org:act:C477A5DF-BA71-4B4D-B788-DC789857BC6D

http://species-id.net/wiki/Zuphioides

Zuphium of authors; (not Latreille 1805 ). Dejean 1831: 298. Chaudoir 1863 : 313. Chaudoir 1872: 103. Putzeys 1878 : 55. LeConte 1879: 62. Horn 1881 : 149. LeConte and Horn 1883: 141. Bates 1883 : 166. Bates 1891: 266. Blatchley 1910 : 139, 140. Schaeffer 1910: 396. Leng 1920 : 65. Csiki 1932: 1562. Liebke 1933 : 461. Darlington 1934: 128. Darlington 1935 : 213. Blackwelder 1944: 70. Hatch 1953 : 150. Ball 1960: 162. Reichardt 1977 : 449. Lindroth 1969: 1089. Erwin 1979a : 360. Erwin 1979b: 564. Kirk 1969 : 17. Erwin 1981: 189, 205. Mateu 1981 : 111. Erwin 1991: 42. Mateu 1993 : 490. Bousquet and Larochelle 1993: 283. Downie and Arnett 1996 : 193. Lorenz 1998: 479 (in part). Peck and Thomas 1998 : 25. Ciegler 2000: 127. Ball and Shpeley 2000 : 397. Ball and Bousquet 2001: 40, 61, 115. Larochelle and Lariviére 2003 : 514 515. Lorenz 2005: 506 (in part). Erwin et al. 2012 : 32. Bousquet 2012: 1355.

##### Type species.

*Zuphium mexicanum* Chaudoir, 1863 (here designated).

##### Generic name.

A compound Latinized noun, treated as neuter, from the generic name *Zuphium* and *oides*, resembling; hence meaning “resembling *Zuphium”.*

##### Recognition.

With character states of Western Hemisphere *Zuphium* genus-group, restricted as follows: size small, pronotum and dorsal surface of elytra densely setose, setae decumbent, body depressed, integument piceous to rufotestaceous, head capsule posteriorly relatively broad, laterally broadly rounded, antennae elongate, antennomere 1 as long or longer than antennomeres 2–4. Humeri broadly rounded. Metasternum long, metepisternum longer than wide at base. Macropterous. Male genitalia: phallus without dorsal paraostial sclerites (Fig. 10D–10F; cf. Fig. 13A–C, **ps**). Ovipositor: Gonocoxite 2 (Figs 11D–F; cf. Figs 11A–C, **gc2**) short, thick; in lateral aspect falciform, apex pointed; in dorso-ventral aspect, broad, paddle-like, apex broadly rounded, not notched. Female genital tract: without secondary spermathecal gland (Fig. 12B; cf. Fig. 13D, **ssg**).

##### Description.

None required here. See description of *Zuphioides mexicanum*, below.

##### Habitat.

Members of this genus are mesophilous to hygrophilous, occupying wet meadows and flood plains principally in open sites, but also in shaded areas along streams, in tropical gallery forest. For more details, see Erwin (1991: 42), Erwin et al. (2012: 32), and Larochelle and Lariviere (2003: 514–515). Ball and Shpeley (2000: 397) mistakenly classified *Zuphium* as “xerophilous”, in part.

#### Zuphioides mexicanum

(Chaudoir)

http://species-id.net/wiki/Zuphioides_mexicanum

Figs 1B , 2B , 3B , 4C, 4D , 5C, 5D , 7A–7F , 8D–8F , 9A , 10D–10F , 11D–11F , 12B

Zuphium mexicanum Chaudoir, 1862: 314. Bates 1883 : 166. Mateu 1981: 125.
Zuphium vicinum Liebke, 1933: 471. Mroczkowski 1960 : 400. Mateu 1985: 329.

##### Type material.

For *Zuphioides mexicanum*, two females, Oberthür-Chaudoir Collection, in front of the following box label: “mexicanum/ Chaud/ Mexique/ 57. Sallé”. LECTOTYPE female, labeled as follows: “33”/ “Ex Musaeo/Chaudoir” [red print]; “Lecto/type” [circular, ringed with red]; in Museum National d’Histoire Naturelle, Paris; PARALECTOTYPE labeled “ Ex Musaeo/Chaudoir” [red print]. Designated by Mateu (1985: 330). For *Zuphium vicinum*, holotype, in Institute of Zoology, Polish Academy of Sciences, Warsawa, #1711.

##### Type area.

Indicated by Mateu (1985: 330) as Veracruz, for *Zuphioides mexicanum*; for *Zuphium vicinum*, “Mexico”.

##### Specific epithet.

A Latinized eponym, nominative case, based on the name of the country in which the type locality is located.

##### Description.

*Size and proportions*. Small, OBL 4.88–4.96 mm; EW 1.60–1.62 mm A1L/A2–4L 0.90–1.00; HW/PW 0.92–0.97. (For seven species of *Zuphioides*: OBL 4.64–6.586 mm; EW 1.60–1.84 mm A1L/A2–4L 0.88–1.12; HW/PW 0.91–0.97).

*Color*. Body rufotestaceous; appendages slightly paler; vestiture testaceous.

*Habitus* (Fig. 1B). Flat, overall. Head capsule (dorsal aspect, Fig. 2B) trapezoidal, obtusely angulate posteriolaterally.

*Eyes* (Figs 3B). Convex, easily seen, ommatidia clearly evident at 50× (about 16 ommatidia are crossed on a horizontal diameter).

*Pronotum*. Narrow.

*Elytra* (Fig. 1B). Lateral margins straight, not bowed, parallel to one another.

*Microsculpture*. Dorsal surface of head capsule (including clypeus) and pronotum smooth, microlines not evident; labrum and elytra with microlines fine (not easily seen at 50× or lower magnification), mesh pattern isodiametric. Ventral surface with microlines fine, generally transverse.

*Luster*. Dorsal surface of head and pronotum shiny, elytra somewhat duller.

*Body vestiture and punctation*. Dorsal surface of head and pronotum sparsely punctate, vestiture sparse. Elytra and abdominal sterna III-VII with punctation and vestiture dense, vestitural setae decumbent.

*Fixed setae*. Head (Fig. 4C): clypeus one pair; head capsule with anterior pair of supraorbitals (**asos**) above eyes; posterior pair of supraorbitals (**psos**) posteriad eyes; one pair of postocular setae (**pos**) immediately behind eyes, laterally; one pair of occipital setae (**ocs**) posteriorly and mediad eyes; posterior supernumerary setae lacking. Antennomere 1 (Figs 4B, 4D, **as1**) with single long seta distally, in addition to decumbent vestiture and row of small erect setae (**as2**, **as3**). Pronotum with two pairs of lateral marginal setae, anterior pair in anterior 1/8, posterior pair at posteriolateral angles. Prosternum with one pair setae anterioventrally (Fig. 9B). Elytra: each elytron anteriorly with parascutellar seta; lateral setae about 21 (one group anteriorly, one group posteriorly, and single seta medially. Abdominal sterna IV-VI without evident ambulatory setae, sternum VII with pair of long setae near posterior margin.

*Head*, *dorsal aspect* (Fig. 4C). Occiput posteriorly and postocciput markedly constricted, in form of narrow neck, postoccipital suture evident. Frontoclypeal suture present. Genal sulcus (**gs**) prominent, but less so than in *Coarazuphium* (cf. Fig. 4A).

*Eyes* (Fig. 4B). Macrophthalmous, markedly convex, readily seen, ommatidia evident at 50×, or lower magnification.

*Antennae* (Fig. 4D). Antennae filiform, extended about body length. Antennomere 1 rather slender, slightly longer than antennomeres a2-a4; antennomere 2 short, about half length of a3; antennomeres 3-11 narrow, cylindrical, distinctly longer than wide.

*Mouthparts*. As described for *Zuphium* genus-group, above. *Labrum* (Fig. 5C). Anterior margin concave. Epipharynx (Fig. 5D). *Mandibles* (Figs 7A–7F). *Maxillae* (Figs 8D, 8E). *Labium*, *ventral aspect* (Fig. 8F).

*Pronotum* (Fig. 1B). Anterior margin truncate, lateral margins markedly sinuate posteriorly, posteriolateral angles prominent, dentiform, slightly anteriad posterior margin; surface impressions (anterior and posterior transverse and median longitudinal) shallow; lateral grooves and posteriolateral impressions moderately deep.

*Pterothorax*. Metasternum of average length; metepisternum elongate, lateral margin longer than anterior margin.

*Elytra* (Fig. 1B). Separate from one another, not fused along suture. Each elytron more or less rectangular, lateral margin straight; humerus projected anteriorly, basal ridge straight; apical margin truncate, with narrow band of membrane. Surface with striae very shallow, intervals almost flat.

*Hind wings*. Macropterous; wings folded beneath elytra at rest.

*Legs*. Male fore-tarsus (Fig. 9A).

*Abdomen*. As described for Western Hemisphere *Zuphium* genus-group.

*Male genitalia* (Figs 10D–10F). Phallus narrow (PW/PL 0.254), slightly curved ventrally, narrowed apically, apical margin rounded, apical portion very short (AL/PL 0.036), ostial membrane extensive (OM/PL 0.317). Left paramere (**lp**) conchoid. Right paramere (**rp**) short, ovoid in form (RP/LP 0.317).

*Female genitalia*: *ovipositor* (Figs 11D–11F). Gonocoxite 1 (**gc1**) with patch of long trichoid setae distally on ventral surface. Gonocoxite 2 (**gc2**) short, thick; in lateral aspect falciform, apex pointed, row of four long trichoid setae dorso- and ventro-laterally; in dorso-ventral aspect, broad, paddle-like, apex broadly rounded, not notched; otherwise, as described for Western Hemisphere *Zuphium* genus-group.

*Female genital tract* (Fig. 12B). Bursa copulatrix (**bc**) bulbous (collapsed in Fig. 12B), inserted at base of common oviduct (**co**). Spermatheca (**sp**) inserted near junction of common oviduct and bursa copulatrix, markedly elongate and slender, with helminthoid sclerite (**hs**) at base. Spermathecal gland duct inserted near base of spermatheca, with distinct swelling proximad spermathecal gland (**spg**). Without secondary spermathecal gland.

##### Geographical distribution.

This species ranges from northern Mexico (states of Nuevo Leon, Nayarit, and Tamaulipas) northward to southwestern U.S.A (states of Arizona, New Mexico, and Texas; Bousquet (2012: 1357).

## Concluding remarks

Treatment of the Western Hemisphere zuphiines remains incomplete. The large geographical gap between the Brazilian and Mexican species of *Coarazuphium* causes one to wonder just how large the gap is. Surely, considering that this genus is likely to be an old (early Tertiary) resident of the Middle American highlands, we can expect therein additional species.

At hemisphere level, *Zuphioides* must be reviewed in detail, using as the principal basis the work of Joaquin Mateu (1981). No doubt, many more species remain to be discovered and described. From a World perspective, a generic-subtribal revision of the Zuphiini is required, based primarily on structural details of the male and female reproductive organs.

## Supplementary Material

XML Treatment for Coarazuphium

XML Treatment for Coarazuphium whiteheadi

XML Treatment for Zuphioides

XML Treatment for Zuphioides mexicanum

## Appendix I

### Tribe Zuphiini in the Western Hemisphere Nature of the Zuphiini

Based on morphological features, most authors have treated the Zuphiini as a “natural” group, meaning in phylogenetic parlance that it is essentially monophyletic. But a molecular analysis, based on two nuclear genes (28S rDNA and *wingless*) conducted by Ober and Maddison (2008: 8, fig. 2) indicated otherwise, namely that this tribe is polyphyletic, its constituent genera having emerged on four different lines, though all of these are within the supertribe Zuphiitae. However, the Ober/Maddison sample included only a small part of zuphiine diversity, and sequences from only two genes. The limited taxon sampling is especially problematic given the large divergences between DNA sequences within Zuphiitae. In addition, the bootstrap values for this result were low (Ober and Maddison 2008: 6, fig. 1). We choose to continue treating the tribe as a practical taxonomic unit, with the hope that a more extensive sample of both zuphiite taxa and genes will give a different result, namely confirming the monophyly of the morphologically based Zuphiini.

### Recognition

Specimens of this tribe are small in size (overall length less than 7.00 mm), integument various (nearly white, testaceous to piceous), and generally pilose, head more or less constricted posteriorly, antennomere 1 (scape) elongate more than length of antennomeres 2+3 and wider than antennomeres 2-11; elytra with apical margin truncate, or subtruncate-sinuate, with a narrow membranous fringe; elytral interval 3 without fixed setae; tibiae without prominent spines. For a more detailed characterization of Zuphiini, see Jeannel (1942: 1091–1092).

### Geographical distribution

The range of this tribe in the Western Hemisphere is co-extensive with the range of the *Zuphium* genus-group, as noted above.

### Classification

Based on previous studies, Reichardt (1977: 448) included five Western Hemisphere genera in the tribe Zuphiini, which he arranged in two subtribes: Zuphiina (*Zuphium* Latreille, 1805) and Patriziina (*Pseudaptinus* Laporte de Castelnau, 1835; *Thalpius* LeConte, 1851; *Mischocephalus* Chaudoir, 1862; and *Metaxidius* Chaudoir, 1852). A third subtribe (Leleupidiina), with genera known only from the Old World tropics, was recognized but not treated further. To these, Mateu (1992) added the monogeneric Mischocephalina, placing therein the Neotropical *Mischocephalus* Chaudoir. Previously, Mateu (1982) had added the Neotropical monobasic genus *Chaudoirella* Mateu, including it in the Patriziina.

Lorenz (2005: 504–507) recognized only three subtribes, namely Leleupidiina, Dicrodontina, (a monogeneric group proposed by Machado (1992: 569)), and Zuphiina, including in the last-named the Zuphiina of authors, Patriziina, and Mischocephalina. We accept provisionally the Lorenz proposal of three subtribes, but recognize genus-groups within the Zuphiina. For the Western Hemisphere, we recognize the *Zuphium* genus-group, the *Patrizia* genus-group (the latter name proposed by Alluaud 1931, a junior synonym of *Agastus* Schmidt-Goebel, 1846), and, the *Mischocephalus* genus-group. Based on marked similarities in the female genitalia, we place *Chaudoirella* (Mateu 1992: 196, fig. 1H; cf. fig. 199, fig. 3A) in the *Mischocephalus* genus-group. We note also that the marked divergence in structure of the male genitalia and female ovipositor between the members of *Agastus* and those of *Pseudaptinus* + *Thalpius* suggest that the latter might best be placed in a supraspecific group of their own.

The following key distinguishes among the Western Hemisphere genus-groups and genera of Zuphiini. Note that among other recent authors, Messer (2011: 419-424) and Bousquet (2012: 1359) treat *Thalpius* and *Pseudaptinus* as subgenera of genus *Pseudaptinus*. Messer (2011) provides a key to the Mexican-USA species of *Thalpius*.

Martinez (2005: 426–434, figs 3.132–3.136) provides a habitus illustration for the following genera: *Metaxidius*, fig. 3.132; *Mischocephalus*, fig. 3.133; *Pseudaptinus*, fig. 3.134; *Thalpius*, fig. 3.135; and *Zuphium* (*auct.*), fig. 3.136. A habitus illustration of *Chaudoirella* is found with the original description (Mateu 1982: 48). Habitus illustrations of each of the Brazilian species of *Coarazuphium* are found with their respective original descriptions.

### Key to Western Hemisphere genera of Tribe Zuphiini (modified from Reichardt (1977: 444) and Mateu (1992: 195–196))

**Table:**

1	Maxillary and labial palpi similar to one another in size and proportions; maxillary palpomere 4 (Figs 8A–8F) not markedly enlarged. Head posteriorly markedly constricted as a narrow neck (Figs 1A, 1B). Male fore-tarsomeres 1-3 with adhesive vestiture pad-like, not biseriate (Fig. 9A) or with a single row of squamo-setae	*Zuphium* genus-group, 2
1’	Maxillary palpi much larger than labial palpi; maxillary palpomere 4 markedly enlarged. Head posteriorly less constricted. Male fore-tarsomeres 1-3 with adhesive vestiture biseriate, or a single row of squamo-setae	3
2(1’)	Eyes absent or flat, ommatidia not evident. Metepisternum about quadrate. Elytra with humeri markedly constricted, lateral margins broadly rounded (Fig. 1A)	*Coarazuphium* Gnaspini, Vanin & Godoy
2’	Eyes convex, normal size, ommatidia evident. Metepisternum elongate, longer than wide at base. Elytra with humeri broadly rounded, lateral margins straight, parallel to one another (Fig. 1B)	*Zuphioides*, gen. n.
3(2’)	Head posteriorly constricted as very narrow neck (more or less as wide as diameter of one eye (cf. Fig. 1A)). Gonocoxite 2 of ovipositor without ensiform setae, with slender setae on dorsal margin, near base (Mateu 1992: 196, fig. 1I; 199, fig. 3A)	*Mischocephalus* genus-group, 4
3’	Head posteriorly with neck thick (wider than diameter of one eye). Gonocoxite 2 with broad ensiform setae (Mateu 1992: 201, figs 4A, 4B)	*Patrizia* genus-group, 5
4(3)	Elytron with lateral margin straight for most of length. Pronotal posteriolateral angles long, very prominent spines. Labium with submentum and mentum fused (Mateu 1992: fig. 1G)	*Mischocephalus* Chaudoir
4’	Elytron with lateral margin broadly rounded. Pronotal posteriolateral angles short spines. Labium with submentum and mentum separated by a suture	*Chaudoirella* Mateu
5(3’)	Pronotal posteriolateral angles not spined	*Pseudaptinus* Laporte de Castelnau
5’	Pronotal posteriolateral angles each a small, sharp spine	6
6(5’)	Antennal socket limited above and below by a sharp carina, inferior carina better developed than superior one (clearly visible from above). Antennomere 1 relatively shorter (slightly shorter than antennomeres 2-4). Pronotum as long as wide; anterior angles more or less sharp. Integument generally glabrous	*Metaxidius* Chaudoir
6’	Antennal socket with superior and inferior carinae equally developed. Antennomere 1 relatively longer (as long or longer than antennomeres 2-4). Pronotum longer than wide. Pronotum and elytra setose	*Thalpius* LeConte
